# Factors affecting the choice of treatment center by infertile couples: A cross-sectional study in Yazd Reproductive Sciences Institute

**DOI:** 10.18502/ijrm.v21i10.14538

**Published:** 2023-11-24

**Authors:** Mohammad Ranjbar, Ali Mohammad Abdoli, Tahereh Shafaghat, Hasan Jafari, Golnaz Izadpanah, Yibeltal Assefa

**Affiliations:** ^1^Health Policy and Management Research Center, School of Public Health, Shahid Sadoughi University of Medical Sciences, Yazd, Iran.; ^2^Yazd Reproductive Sciences Institute, Shahid Sadoughi University of Medical Sciences, Yazd, Iran.; ^3^School of Public Health, Faculty of Medicine, University of Queensland, Brisbane, Australia.

**Keywords:** Infertility, Fertility clinics, Yazd, Iran.

## Abstract

**Background:**

Infertility is one of the critical health issues in Iran. There are more than 70 specialized infertility treatment centers in Iran, of which the Yazd Reproductive Sciences Institute, is one of the most important ones.

**Objective:**

This study aimed to determine the factors influencing infertile couples' choice of Yazd Reproductive Sciences Institute.

**Materials and Methods:**

This cross-sectional study was conducted on 275 infertile couples aged 18 and older, referring to Yazd Reproductive Sciences Institute, Yazd, Iran from September 2021 to March 2022. Data were gathered using a 2-part questionnaire. Data analysis was done through SPSS software. We used descriptive statistics, Kruskal-Wallis, Mann-Whitney, and *t *test for data analysis.

**Results:**

Most participants were individuals who came from other provinces of Iran (74.9%) and were referred to the Yazd Reproductive Sciences Institute. Among the 4 categories that influenced couples' decision to choose this center, factors related to the personnel and treatment staff received the highest score (75.83), while personal factors received the lowest score (65.76). The average score for factors related to doctors was 72.90, and for factors related to the center, it was 73.65. The satisfaction with personnel and treatment staff varied based on participants' education levels, with those who had lower levels of education reporting higher levels of satisfaction (p < 0.001).

**Conclusion:**

The primary factors contributing to the success of the Yazd Reproductive Sciences Institute in attracting clients were the dedication and expertise of the staff, as well as the esteemed reputation of the doctors at the center.

## 1. Introduction

Infertility is known to be one of the worldwide concerns of the 21 ${}^{\text{st}}$ century (1, 2). The evidence reports the prevalence of 8-15% of infertility among the world's population (3-5). While in some regions, the infertility rate reaches about 30% (6). According to some studies in Iran, the infertility rate is 8-11% in the population (7, 8). There are different definitions for infertility. According to the World Heath Organizations definition, infertility refers to the inability to attain a clinical pregnancy after engaging in regular unprotected intercourse for a period of 12 months (9).

Approximately 56% of infertile couples seek medical treatment, leading to a rise in cross-border fertility care where individuals travel to other locations for desired infertility treatments (4). This has contributed to an increase in the number of infertile couples seeking international facilities for infertility treatments due to the growth of medical tourism (10, 11).

Iran has over 70 clinics and specialized medical centers offering infertility treatments, indicating a competitive market in the country (12). Client satisfaction is crucial for the success and sustainability of healthcare centers, prompting medical centers to assess client expectations in order to attract more participants, reduce costs, and increase income (13).

When choosing a treatment center, infertile couples prioritize effective communication and quality services, with the success rate being a significant factor in their decision-making process (14, 15). Other factors include doctors' recommendations, geographical distance, and factors influencing the selection of infertility treatment centers (16). Yazd province has made significant advancements in infertility treatment, making it one of the leading regions in Iran for such treatments (17).

The province has the potential to attract international patients, particularly in the field of infertility treatment. This study aims to identify the factors that influence the selection of the Yazd Reproductive Sciences Institute by participants, which will help improve the quality of the center and attract more infertile couples.

## 2. Materials and Methods

This cross-sectional study was conducted on 275 infertile couples aged 18 and older, referring to Yazd Reproductive Sciences Institute, Yazd, Iran from September 2021 to March 2022.

The criteria for inclusion in the study were being a first-time visitor to the center and not having previously used their medical services. Participants who chose not to complete the questionnaire for any reason were excluded from the study.

The couples completed the questionnaire together and returned it to the researchers, which is why the gender variable was not included in the analysis. In cases where participants were unable to read or write or had difficulty completing the questionnaire, the researchers assisted in filling it out based on their responses.

To reduce potential data bias, participants were assured that their participation in the study would not have any impact on the quality of services they may receive in the future.

Data collection was done by a 2-part questionnaire that was initially used by Varmaqani et al. (18).

The face and content validities of the questionnaire were re-evaluated by 10 experts in health management and infertility treatment. The reliability of the questionnaire was assessed using Cronbach's alpha coefficient. In terms of content validity, 22 out of the 29 questions in the questionnaire received a content validity ratio score of 0.80, while the remaining 7 questions received a score of 1. According to the Lawshe formula and input from the experts, a minimum acceptable score of 0.62 was established. Since all the questions met or exceeded this threshold, no modifications were necessary. The content validity index was determined to be 84.42.

The final questionnaire consisted of 6 questions about the attending physician, 5 questions about the treatment staff and other personnel, 11 questions about personal and other factors, 7 questions about the treatment center, and 2 double-choice questions (yes or no). Additionally, there were 2 open-ended questions asking participants about the important factors in selecting a treatment center.

The questions were scored using a Likert scale, with very high and high assigned a score of 3, average assigned a score of 2, and low and very low assigned a score of 1. Therefore, the total scores for this questionnaire ranged from 29-145.

To determine the reliability of the questionnaire, Cronbach's alpha coefficient was used. The questionnaire was distributed among 30 individuals who were referred to and admitted to the infertility treatment center. After they answered the questionnaire, it was collected. The pilot study showed Cronbach's alpha coefficient of 0.87, indicating sufficient reliability of the questionnaire.

### Sample size

Participants were selected by a simple random sampling method, based on the Cochran sample size formula. 


$$n=\frac{\frac{z^{2}pq}{d^{2}}}{1+\frac{1}{N}\left[\frac{z^{2}pq}{d^{2}}-1\right]}$$

Considering the values of α = 0.05, σ = 3.37, and d = 0.4, the minimum number of required samples was calculated to be 275.

### Ethical considerations

This research was approved by the Ethics Committee of Shahid Sadoughi University of Medical Sciences in Yazd, Iran (Code: IR.SSU.SPH.REC.1400.115). All methods were performed in accordance with the relevant guidelines and regulations. Informed oral consent was obtained from all participants.

### Statistical analysis

Data analysis was performed using SPSS version 22.0. Descriptive statistics such as mean, standard deviation, frequency, and percentage were used to report the findings. To examine the relationship between demographic variables and factors influencing the selection of the Yazd Reproductive Sciences Institute, non-parametric tests including Kruskal-Wallis and Mann-Whitney tests were utilized due to the non-normal distribution of quantitative variables. The significance level for these tests was set at 0.05.

## 3. Results

In our study, a total of 275 infertile couples aged 18 and older completed the questionnaire. The average distance between the participants' place of residence and the Yazd Reproductive Sciences Institute was 6.6 ± 5.16 hr, with a maximum distance of 30 hr. The number of couples who used their personal or rented vehicle to reach the treatment center was equal. The majority of participants (38.5%) chose to go to the Yazd Reproductive Sciences Institute based on recommendations from friends and acquaintances. A significant portion of the participants (74.9%) came from provinces other than Yazd. Specifically, 15.3% were from Yazd province, and 9.8% lived in Yazd city.

Regarding health insurance coverage, most participants (94.1%) had insurance, with 67.9% covered by social security insurance. The majority (68.2%) did not have any supplementary insurance. In terms of education, 41.2% of participants had diploma or associate degrees. Self-employment was the most common occupation among participants, with 51% identifying as self-employed. The majority of participants (51%) reported a monthly income level between $100 and $200.

The participants' demographic characteristics are displayed in table I. The factors related to personnel and treatment staff received the highest score among the 4 domains influencing the referral of individuals to the Yazd Reproductive Sciences Institute. The scores for each factor affecting the selection of infertility treatment centers can be found in table II.

A significant association was found between education level and job type and the factors influencing the selection of infertility treatment centers within the personnel domain. Individuals with a high school education level or below considered personnel-related factors more influential in choosing the Yazd Reproductive Sciences Institute (p < 0.001). Similarly, those with self-employed occupations rated personnel-related factors higher in their choice of treatment center compared to individuals with other occupations (p = 0.01). However, there were no significant differences in scores across other domains based on participants' education levels (Table III).

Table III also reveals a significant relationship between income level and the scores of the physician and clinical staff domains. Couples with a monthly income of less than $100 (Each US dollar in 2021-2022 was roughly equivalent to 250,000 Rials) considered factors related to physicians (p = 0.04) and personnel (p = 0.04) more important when selecting a treatment center.

According to the findings, couples insured with Iranian health insurance and social security insurance placed greater importance on factors related to the treatment center when choosing the Yazd Reproductive Sciences Institute compared to those insured with other basic insurances (p < 0.001). Additionally, individuals covered by Iranian health insurance assigned higher scores to personal factors in their selection of the center compared to couples covered by other insurance (p < 0.001). However, no correlation was observed between income level and scores for factors related to the center and personal factors.

**Table 1:** Demographic characteristics (n = 275)


**Variables**		**Mean ± SD**	**Frequency (N %)**
**Age (yr)**		32.8 ± 6.3	-
**Time distance (hr)**		6.6 ± 5.16	-
**Type of accommodation**			
	**Owner**	- 129 (47.3)
	**Tenant**	- 129 (47.3)
	**Other**	- 15 (5.4)
**Insurance status**			
	**Covered by insurance**	- 255 (94.1)
	**Without insurance**	- 15 (5.9)
**Basic insurance type**			
	**Iranian health insurance**	- 50 (18.7)
	**Social security insurance**	- 182 (67.9)
	**Other**	- 36 (13.4)
**Supplementary insurance**			
	**Covered by supplementary insurance**	- 64 (31.8)
	**No supplementary insurance coverage**	- 137 (68.2)
**Education**			
	**High school and below**	- 71 (26.1)
	**Diploma and associate degree**	- 112 (41.2)
	**Bachelor's degree or higher**	- 89 (32.7)
**Income level (US Dollar)**			
	**< 100**	- 75 (27.9)
	**100-200**	- 106 (39.4)
	**> 200**	- 88 (32.7)

**Table 2:** Factors affecting the selection of the Yazd Reproductive Sciences Institute in 2022


**Factors**	**Mean ± SD**
**Physician **	72.90 ± 18.97
**Personnel **	75.83 ± 17.75
**Center **	73.65 ± 16.79
**Personal **	65.76 ± 12.84

**Table 3:** The relationship between demographic variables and factors affecting the selection of the Yazd Reproductive Sciences Institute


**Variables**		**Physician related factors**	**Personnel related factors**	**Center related factors**	**Personal related factors**
**Education level**					
* *	**High school and below**	75.74 ± 16.22	79.94 * ± * 17	72.87 * ± * 13.55	66.39 * ± * 12.36
	**Diploma and associate degree**	74 * ± * 22.05	75.63 * ± * 14.59	74.78 * ± * 12.54	66.54 * ± * 13.19
	**Bachelor's degree or higher**	69.18 * ± * 16.80	72.76 ± 21.37	72.90 ± 22.66	64 ± 12.89
	**P-value**	0.06	0.00	0.24	0.40
**Job type**					
	**Employee**	70.24 * ± * 14.96	69 ± 16.60	70.17 ± 12.79	63.56 ± 9.97
	**Self-employment**	75.53 * ± * 20.28	77.78 ± 15.73	75.38 ± 19.12	66.43 ± 12.72
	**Student**	70.47 * ± * 13.57	66.50 ± 21.46	65.89 ± 15.45	58.48 ± 16.80
	**Other**	69.85 * ± * 18.03	77.36 ± 20.21	72.85 ± 14.13	65.47 ± 13.01
	**P-value**	0.20	0.01	0.11	0.38
**Income level (Million Rials)**					
	**< 20**	78.28 ± 23.52	79.89 ± 14.75	74.53 ± 12.39	67.56 ± 13.08
	**20-40**	70.13 ± 16.87	74.70 ± 16.83	73.44 ± 21.44	65.47 ± 13.37
	**> 40**	71.56 ± 16.68	73.95 ± 20.86	73.23 ± 13.86	64.77 ± 12.26
	**P-value**	0.04	0.04	0.64	0.40
**Insurance type**					
	**Iranian health insurance**	74.59 ± 15.87	79.51 ± 16.54	75.90 ± 12.03	70.68 ± 12.07
	**Social security insurance**	72.74 ± 20.62	74.64 ± 16.11	74.41 ± 18.24	65.72 ± 12.63
	**Other**	71.56 ± 16.08	75.66 ± 26.22	66.53 ± 13.53	58.73 ± 12.21
	**P-value**	0.74	0.13	0.00	0.00
Data presented as Mean ± SD. Kruskal-Wallis for non-parametric and Mann-Whitney test for parametric variables					

## 4. Discussion

According to the findings of this study, participants identified the personnel and treatment staff as the most influential factor in their selection of an infertility treatment center in Yazd City. This is consistent with the reputation of the city for having compassionate individuals, which is reflected in the staff at the infertility treatment center. Similar studies conducted in other cities such as Rasht and Tehran also found that factors related to the treating physician and clinical staff were highly influential in choosing a treatment center (18-20). A study also reported economic factors, hospital equipment, and facilities as the most effective factors in choosing a hospital (20).

The participants in this study identified several key features of the Yazd Reproductive Sciences Institute that influenced their choice of the center. These included personal desire, confidentiality, honesty of the treatment staff, the center's history and reputation, the cost of treatment, cleanliness and hygiene, reputation of the doctors, discipline of the center, presence of experienced doctors, and advanced treatment equipment and facilities.

The Yazd Reproductive Sciences Institute holds significance in Iran as the country's first infertility treatment center, established in 1989. The center's reputation, particularly due to renowned figures like Professor Abbas Aflatoonian, plays a major role in attracting infertile couples. Previous studies conducted in Iran have also highlighted the importance of the physicians' skill and reputation in clients' choice of an infertility treatment center (19, 21).

Referrals from friends and acquaintances, as well as individual opinions, were common methods of selecting the treatment center among the participants. The nature of required services, such as inpatient or outpatient care, and emergency or elective treatments, also influenced the choice of treatment center (21, 13).

Interestingly, a significant proportion of participants who sought treatment at the Yazd Reproductive Sciences Institute were from outside Yazd province, indicating its popularity among infertile tourists.

The study found a relationship between income level and the importance placed on factors related to physicians and personnel. Individuals with lower income prioritized factors such as physician behavior and skill over center-related factors like discipline, cleanliness, and advanced equipment. Education level also influenced participants' perceptions of personnel-related factors. Those with a high school education or below placed greater importance on the behavior, commitment, and competence of the center's employees compared to other factors. This finding aligns with a previous study that found individuals with higher education levels valued infrastructure factors like up-to-date equipment more (13). However, another study reported that individuals with higher education levels considered external factors and quality to be more important than internal issues like personnel skill and behavior when choosing an infertility treatment center (22). Based on the results of our study, we found that insured participants under the coverage of Iranian Health Insurance and Social Security Insurance, the 2 main basic insurances in Iran, placed significant importance on center-related factors when choosing the Yazd Reproductive Sciences Institute compared to other basic insurance policyholders. This difference may be attributed to the more limited coverage of costs in Iranian Health Insurance and Social Security Insurance compared to other basic insurances such as Armed Forces and Banks Health Insurance. The limited coverage has made center-related factors, especially those related to service costs, more important for patients under Iranian Health and Social Security basic insurances. Therefore, these patients have chosen the government-affiliated Yazd Reproductive Sciences Institute for receiving infertility treatment services because it offers government tariffs.

Insurance coverage greatly impacts the cost of services, and more extensive coverage leads to reduced financial concerns among patients (21). This finding is consistent with studies conducted in Oman and Ghana, which have also confirmed the relationship between basic insurance status and type with the influencing factors in choosing an infertility treatment center (23, 24).

## 5. Conclusion

In conclusion, our study highlights that work commitment and experience among employees, as well as the reputation of physicians working at the Yazd Reproductive Sciences Institute, are critical reasons for its success in attracting infertile couples. Financial issues were also identified as one of the most important factors in choosing this center, particularly due to the affordability of infertility treatment after being covered by insurance. Therefore, we recommend that the center invest in its other competitive advantages and develop a serious advertising plan to further promote its services.

##  Conflict of Interest

The authors declared that there is no conflict of interest.
